# Exploring the Role of Foveal and Extrafoveal Processing in Emotion Recognition: A Gaze-Contingent Study

**DOI:** 10.3390/bs15020135

**Published:** 2025-01-26

**Authors:** Alejandro J. Estudillo

**Affiliations:** Department of Psychology, Bournemouth University, Poole House Talbot Campus, Poole BH12 5BB, UK; aestudillo@bournemouth.ac.uk; Tel.: +44-01202-962229

**Keywords:** emotion recognition, foveal vision, extrafoveal vision, eye tracking, gaze-contingent paradigm

## Abstract

Although the eye-tracking technique has been widely used to passively study emotion recognition, no studies have utilised this technique to actively manipulate eye-gaze strategies during the recognition facial emotions. The present study aims to fill this gap by employing a gaze-contingent paradigm. Observers were asked to determine the emotion displayed by centrally presented upright or inverted faces. Under the window condition, only a single fixated facial feature was available at a time, only allowing for foveal processing. Under the mask condition, the fixated facial feature was masked while the rest of the face remained visible, thereby disrupting foveal processing but allowing for extrafoveal processing. These conditions were compared with a full-view condition. The results revealed that while both foveal and extrafoveal information typically contribute to emotion identification, at a standard conversation distance, the latter alone generally suffices for efficient emotion identification.

## 1. Introduction

Facial expressions are important biological and social cues that have evolved to facilitate communication among peers (Schmidt & Cohn, 2001; F. W. Smith & Schyns, 2009; Susskind et al., 2008). Humans use facial expressions to transmit information about their feelings, intentions, and the environment. For this reason, the accurate and rapid interpretation of a conspecific’s facial emotions is crucial for survival and social interaction (Darwin, 1872). Although, under central viewing conditions, the different types of emotions are recognised with a reasonable level of accuracy, even such optimal conditions are not always present when interacting with others. For example, it is common for people to cover specific facial features with items, such as surgical face masks, neck gaiters, or sunglasses, which can dramatically reduce emotion recognition accuracy (Grundmann et al., 2021; Kim et al., 2022; Wong & Estudillo, 2022).

The physical obstructions of facial features are not the only challenge that our visual system encounters in identifying emotions. For example, in daily life, we also frequently perceive faces at different eccentricity from our central vision or extrafoveally. In addition, even when these faces are perceived in front of us at a normal conversation distance, not all the facial features fall within our central vision, and some of these features will be processed extrafoveally. Despite the importance of foveal and extrafoveal information in visual cognition, their independent contributions to emotion recognition remain poorly understood. Thus, in this study, we investigated the independent roles of foveal and extrafoveal information in emotion recognition.

### 1.1. Literature Review

Foveal or central vision refers to the area of the visual field that is preferentially specialised in the processing of fine detail and high-resolution visual information (Stewart et al., 2020). This area, which corresponds approximately up to 2° eccentricity, contains a higher density of cones. Extrafoveal vision refers to the area of the visual field outside foveal vision. This area includes parafoveal vision, which extends approximately from 2° to 4–5° of the visual field, and peripheral vision, which encompasses the rest of the visual field. Visual acuity and contrast sensitivity decline as the eccentricity from foveal vision increases (Larson & Loschky, 2009; Loschky et al., 2005, 2019). Importantly, foveal and extrafoveal vision differ not only quantitatively in terms of acuity and resolution but also qualitatively in visual processing and task optimisation (Atkinson & Smithson, 2020; Duran & Atkinson, 2021; Rosenholtz, 2016; Strasburger et al., 2011). These qualitative differences are particularly critical in face perception research. Studies have shown that face-specific mechanisms are predominantly supported by foveal processing (Canas-Bajo & Whitney, 2022; Goren & Wilson, 2006; but see McKone, 2004). For instance, Canas-Bajo and Whitney (2022) demonstrated that the recruitment of face-specific mechanisms was reduced at 6° eccentricity compared with foveal vision.

Although emotion detection (e.g., discriminating neutral faces from fearful faces) appears to be relatively preserved at an eccentricity of up to 40° compared with gender detection (Bayle et al., 2011), emotion recognition performance generally decreases as the eccentricity from central presentation increases, with this effect being modulated by the type of emotion. For example, a recent study showed that emotion discrimination performance was impaired when the faces were presented at an eccentricity of 15° from central view (F. W. Smith & Rossit, 2018). Similar results were reported by an earlier study with synthetic faces, which revealed that the identification of angry, fearful, and sad face expressions was impaired at an eccentricity of 8° compared with central presentation (Goren & Wilson, 2006). Interestingly, this effect of eccentricity was not observed with happy faces. This finding does not only show that the perception of facial expressions is differentially affected by visual eccentricity depending on the type of emotion, but it also demonstrates that the so-called happy-face advantage (i.e., the better identification of happy faces compared with others; Calder et al., 2000; Calvo et al., 2014) remains robust even under challenging visual conditions.

However, much less is known about the role of parafoveal vision in emotion recognition. In fact, to the best of our knowledge, only two previous studies have explored the role of parafoveal vision in emotion recognition. In Calvo et al. (2010), a target emotional face and a scrambled face were briefly presented side by side, each at an eccentricity of 2.5° from the centre of the screen. After a backward mask, observers were presented with a probe word (e.g., happy), and they had to indicate if this word matched the previously presented face. The authors found that happy faces were identified faster compared with other emotions. Similarly, all emotions except happy faces were impaired when presented at an eccentricity of 2.5° from the centre compared with a central presentation (Calvo et al., 2014).

Altogether, the results of the reviewed studies suggest that emotion recognition is generally impaired when faces are presented extrafoveally (see also Zoghlami & Toscani, 2023). Nevertheless, these studies are not without limitations. For example, in Calvo and colleagues’ studies (Calvo et al., 2010, 2014) observers were asked to match a probe word to the previously presented face, which is an artificial way to assess emotion recognition that differs significantly from how people identify emotions in daily life. In addition, in the reviewed studies parafoveal and peripheral processing were studied by comparing faces presented at a specific eccentricity with those presented at the centre of the screen. Although this is the standard approach to studying extrafoveal processing in other domains such as reading (Balota & Rayner, 1983), perceptual learning (Beard et al., 1995) and object processing (Castelhano & Pereira, 2018), this approach presents two problems. First, from a methodological point of view, such a presentation confounds face position (i.e., eccentric from the screen centre) with the type of information (i.e., parafoveal or peripheral information). This is problematic as presenting faces eccentrically prevents the observers to process the faces by using face-specific mechanisms (Goren & Wilson, 2006).

Further, this eccentric presentation is also problematic from a more ecological point of view. Although there are no doubts that recognising emotions in the periphery has clear evolutionary advantages, in our daily life, emotions tend to be identified when we are directly interacting with others. Different facial features provide different diagnostic values in the recognition of different emotions (Barabanschikov, 2015; Calder et al., 2000; Calvo et al., 2014; Calvo & Marrero, 2009; Schurgin et al., 2014; M. L. Smith et al., 2005). For example, while the mouth seems to be a particularly important feature for identifying happy and surprise expressions, the eyes seem to be more relevant for the identification of sadness and fear (e.g., Calvo et al., 2014; Wegrzyn et al., 2017). Importantly, in face-to-face social interactions, not all the facial features of a conspecific fall within the foveal area, and some features are instead processed parafoveally or even peripherally. In fact, during a typical interaction at approximately 58 cm from an interlocutor, when observers maintain eye contact, the lower part of the face will fall within the parafoveal area, and farther-down features, such as the mouth, will fall within the peripheral area (Atkinson & Smithson, 2020; Mayrand et al., 2023). It is also noteworthy that although all the previously reviewed studies have demonstrated a clear advantage of central vision over parafoveal and peripheral vision in recognising emotions (Bayle et al., 2011; Calvo et al., 2014, 2014; Goren & Wilson, 2006; F. W. Smith & Rossit, 2018), none of these studies has truly isolated central vision. As previously mentioned, foveal or central vision corresponds to only the central 2° of the visual field. Consequently, in these studies, the central presentation of faces incorporates both foveal and extrafoveal information. Thus, the unique contribution of foveal and extrafoveal information to emotion recognition remains unknown.

The eye-tracking technique has been widely used in face-processing research to investigate observers’ gaze behaviour while performing different tasks (Althoff & Cohen, 1999; Bindemann, 2010; Lee et al., 2022; Peterson & Eckstein, 2012; Williams & Henderson, 2007). Although influenced by several factors (Yitzhak et al., 2021), emotion recognition research using eye tracking suggests that different facial expressions are associated with specific fixation patterns (Barabanschikov, 2015; Eisenbarth & Alpers, 2011; Paparelli et al., 2024; Schurgin et al., 2014), while the eye region tends to be fixated more frequently and for longer durations in anger and sadness compared with the mouth (Eisenbarth & Alpers, 2011; Schurgin et al., 2014), the opposite pattern is observed for happy faces (Beaudry et al., 2014; Eisenbarth & Alpers, 2011).

Although informative, using fixation patterns and other eye-tracking measures as dependent variables only provides a descriptive account of the relevance of different parts of the face to identifying emotions. Interestingly, the eye-tracking technique can also be potentially used as an independent variable by manipulating the amount and type of information through gaze-contingent paradigms (Billino et al., 2018; Estudillo & Bindemann, 2017; Hagen et al., 2023; McConkie & Rayner, 1975; Miellet & Caldara, 2012; Van Belle et al., 2010a, 2010b). For example, in Van Belle and colleagues’ study (Van Belle et al., 2010b), observers were first presented with a full-view target face. This face was then followed by two test faces presented side by side, and observers had to indicate which of these two faces corresponded to the target face. The paradigm comprises three experimental conditions. Under the mask condition, an oval mask hides the fixated facial feature but leaves the rest of the face available. In contrast, under the window condition, only the fixated facial feature was visible, forcing observers to rely only in this small area for identification. Both the mask and the window move in a gaze-contingent manner. These two conditions were compared with a control condition, whereby there was not any visual restriction. Compared with the control and mask conditions, performance decreased dramatically under the window condition (Evers et al., 2018; Van Belle et al., 2010a, 2010b; Verfaillie et al., 2014).

### 1.2. The Present Study

By using an adaptation of the gaze-contingent paradigm, the present study aims to dissociate the roles of foveal and extrafoveal information in emotion recognition. Observers were asked to identify anger, fear, happiness, sadness, and surprise facial emotions presented under three conditions: a full-view control condition; a gaze-contingent oval mask condition that blocked foveal information while allowing for extrafoveal information; and a gaze-contingent window condition that blocked extrafoveal information while only allowing for foveal information. Faces were also presented in upright and inverted orientation. This manipulation was included to explore whether the effects of foveal and parafoveal information on emotion recognition are face-specific or reflect more general visual processes (Rossion, 2008; Van Belle et al., 2010b). If foveal information is more important for the recognition of emotions than parafoveal information, we expect to find better performance under the window condition compared with the mask condition. On the contrary, if parafoveal information is more important for emotion recognition, we expect to find better performance under the mask compared with the window condition. Finally, as some research has reported that happy faces are identified with similar levels of accuracy when the faces are presented centrally and parafoveally (Calvo et al., 2010, 2014), the differences between the control and mask conditions might be smaller for happy faces compared with other emotions.

## 2. Materials and Methods

### 2.1. Design

A within-subjects design was utilised. The independent variables were facial emotions, with five conditions: anger, fear, happiness, sadness, and surprise; viewing conditions: full view, masked, and window; and orientation: upright and inverted. The dependent variables were recognition accuracy and response times. To avoid potential speed–accuracy trade-offs, these measures were used to calculate the rate-correct scores (RCSs) (Woltz & Was, 2006), an integrative measure of efficiency to solve cognitive tasks (Vandierendonck, 2017). The RCS is calculated by dividing the number of correct trials by the sum of reaction times for both correct and incorrect trials and represents the number of correct trials per second. Thus, higher RCSs indicate greater efficiency in solving the task.

### 2.2. Participants

Thirty Malaysian Chinese undergraduate students from University of Nottingham Malaysia (females = 15, M_age_ = 20.5 years, SD_age_ = 1.70) took part in this study. Participants reported normal or corrected-to-normal vision.

### 2.3. Stimuli and Apparatus

A total of 20 identities (10 females) were taken from the Taiwanese Facial Expression Image Database (TFEID) (Chen & Yen, 2007). Each of the faces displayed the following emotions: anger, fear, happiness, sadness, and surprise. The external features (e.g., hair and ears) were cropped out of the photographs to direct attention towards inner facial features (e.g., eyebrows, eyes, nose, and mouth). The stimulus images were 480 by 600 pixels (17.93° by 22.41° at a distance of 75 cm from the screen).

The face stimuli were displayed on a 24′ BenQ monitor (driven by Microsoft Windows 7 Professional, version 6.1.7601) with a spatial resolution of 1920 by 1080 pixels. The experiment was programmed on SR Research Experiment Builder (version 1.10.1630). Eye movements were tracked with a desktop-mounted EyeLink 1000+ eye-tracking system at a 1000 Hz sampling rate, positioned 75 cm from the participant. To minimise head movements, participants were asked to place their heads on a chin and head rest.

### 2.4. Procedure

Participants were asked to seat in front of the computer and eye tracker in a dark enclosed room. The chin and head rest and the chair were adjusted for each participant. Verbal and written instructions were given to explain the emotion identification task. Participants were informed to press the response key corresponding with the perceived emotion: ‘d’ for anger, ‘f’ for fear, ‘g’ for happiness, ‘h’ for sadness, and ‘j’ for surprise. The standard nine-dot EyeLink calibration was conducted, and the validation procedure followed.

**Figure 1:**
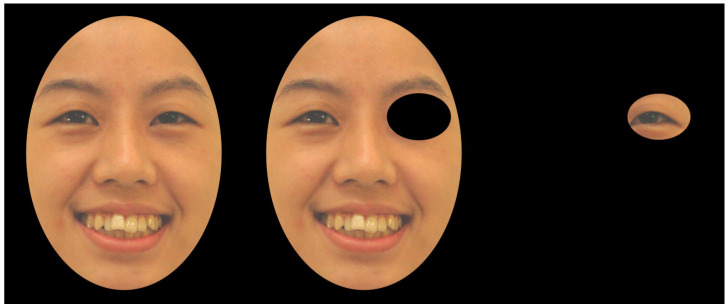
Upright stimuli under the viewing conditions control, mask, and window.

Each trial began with a central drift correction, which was followed by a fixation cross on the left side of the screen. Upon detection of the fixation, a target face appeared in the centre of the screen. The stimuli were presented until response either in full view, with a gaze-contingent mask, or with a gaze-contingent window. Under the mask condition, the fixated face part was covered by an oval mask, but the rest of the face was uncovered. In contrast, under the window condition, only the fixated face part was visible through an oval window. Both the mask and the window were gaze-contingent and measured 127 by 93 pixels (4.75° × 3.62° at a distance of 75 cm from the screen; see Figure 1). A blank screen appeared for 1000ms after the response.

There were 60 trials per emotion. For each emotion, half of the trials were presented in upright orientation, and the other half were presented upside down. For each orientation condition, 10 trials were presented under each of the viewing conditions. Trials were presented in random order, and the allocation of the identities and the emotions to each viewing condition was counterbalanced across participants.

## 3. Results

**Figure 2:**
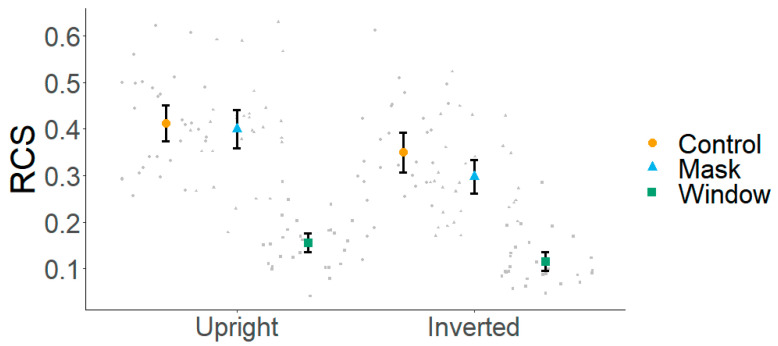
Mean RCSs across viewing and orientation conditions. Error bars denote 95% CIs.

Figure 2 shows the mean RCSs across the orientation and viewing conditions. A 2 (orientation: upright, inverted) × 3 (viewing condition: control, mask, window) repeated measures ANOVA was run. The visual inspection of Q-Q plots indicated that the residuals were normally distributed. When the assumption of sphericity was violated, Greenhouse–Geisser corrections were applied to adjust the degrees of freedom. The ANOVA revealed the main effects of orientation [F(1, 28) = 71.25, *p* < 0.001, η^2^_p_ = 0.71], viewing condition [F(2, 56) = 257.28, *p* < 0.001, η^2^_p_ = 0.90], and an interaction between both factors [F(1.55, 43.40) = 7.41, *p* < 0.01, η^2^_p_ = 0.21]. To explore this interaction, we conducted separate ANOVAs for each orientation. For upright faces, we found a main effect of the viewing condition [F(2, 56) = 192.40, *p* < 0.001, η^2^_p_ = 0.87]. Post hoc analyses (Holm-corrected) revealed similar performance under the control and mask conditions (*p* = 0.38) but better performance under these two conditions compared with the window condition (both *ps* < 0.001, *ds* ≥ 2.68). For inverted faces, the main effect of the viewing condition was also significant [F(2, 56) = 158.10, *p* < 0.001, η^2^_p_ = 0.85]. Post hoc analyses revealed better performance under the control compared with both the mask and window conditions (both *ps* < 0.001, *ds* ≥ 0.58) and under the mask compared with the window condition (*p* < 0.001, *d* = 2.04).

**Figure 3:**
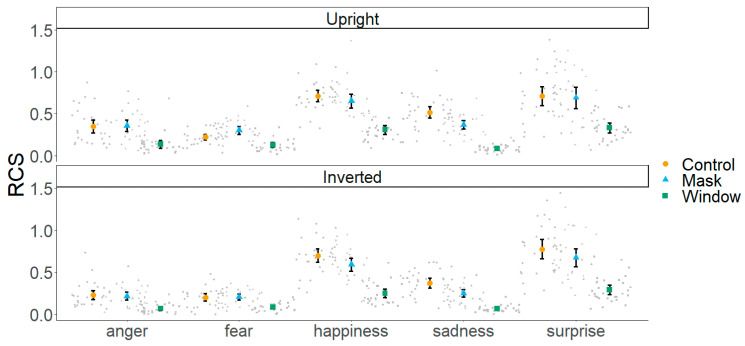
Mean RCSs across emotions, viewing, and orientation conditions. Error bars denote 95% CIs.

As previous research has shown differential roles of central and parafoveal information across different emotions (Calvo et al., 2010, 2014), in the second part of our analysis, we also included the factor emotion (see Figure 3). A 2 (orientation: upright, inverted) × 3 (viewing condition: control, mask, window) × 5 (emotion: anger, fear, happiness, sadness, surprise) repeated measures ANOVA revealed the main effects of orientation [F(1, 28) = 50.20, *p* < 0.001, η^2^_p_ = 0.64], viewing condition [F(2, 56) = 304.77, *p* < 0.001, η^2^_p_ = 0.91], and emotion [F(2.24, 62.94) = 75.49, *p* < 0.001, η^2^_p_ = 0.79]. We also found two-way interactions between orientation and viewing condition [F(2, 56) = 5.62, *p* < 0.01, η^2^_p_ = 0.16], orientation and emotion [F(2.94, 82.35) = 5.13, *p* < 0.01, η^2^_p_ = 0.15], and viewing condition and emotion [F(4.80, 134.61) = 20.97, *p* < 0.001, η^2^_p_ = 0.42]. Finally, the three-way interaction among these factors was also significant [F(8, 224) = 2.87, *p* < 0.01, η^2^_p_ = 0.09].

Based on previous research (Calvo et al., 2010, 2014), we hypothesised that for happy faces, the differences between the control and mask conditions might be smaller compared with other emotions. To explore the three-way interaction, we conducted separate ANOVAs for each emotion (see Table 1). For fear, the ANOVA revealed the main effects of orientation, viewing condition, and interaction between both factors. This interaction seems to reflect better performance under the mask than the control condition in upright trials but similar performance under the control and mask conditions in inverted trials. This pattern was confirmed by separate ANOVAs for upright [F(2, 56) = 38.17, *p* < 0.001, η^2^_p_ = 0.57] and inverted [F(1.50, 42.18) = 34.71, *p* < 0.001, η^2^_p_ = 0.55] trials. For upright trials, a post hoc t-test (Holm-corrected) revealed better performance under the mask than the control or window condition (both *ps* < 0.001, *ds* ≥ 0.80) and better performance under the control compared with the window condition (*p* < 0.001, *d* = 0.94). For inverted trials, performance under the control and mask conditions was better compared with the window condition (both *ps* < 0.001). However, there were no differences between the control and mask conditions (*p* = 0.80, *ds* ≥ 1.31). In addition, separate t-tests for each viewing conditions revealed similar performance for upright and inverted trials under the control condition (*p* = 0.34) but better performance for upright than inverted trials under the mask and window conditions (both *ps* < 0.001, *ds* ≥ 0.65). For sadness, the ANOVA revealed the main effects of orientation, viewing condition, and interaction between both factors. To explore this interaction, we first conducted separate ANOVAs for upright [F(2, 56) = 119.90, *p* < 0.001, η^2^_p_ = 0.81] and inverted trials [F(2, 56) = 78.26, *p* < 0.001, η^2^_p_ = 0.73]. Post hoc analysis revealed that in both upright and inverted trials, participants performed better under the control compared with the masks and window conditions and under the mask compared with the window condition (all *ps* < 0.001, *ds* ≥ 1.53). In addition, separate t-tests for each viewing conditions revealed better performance for upright than inverted faces under the control and mask conditions (both *ps* < 0.001, *ds* ≥ 0.82) and under the window condition, but this effect seems somewhat smaller in the latter (*p* < 0.001, *d* = 0.40).

For anger, happiness, and surprise, the main effects of the viewing condition were significant. A post hoc t-test (Holm-corrected) revealed better performance under the control than the mask condition for happiness (*p* < 0.01, *d* = 0.45) but similar performance across these conditions for anger and surprise (both *ps* ≥ 0.09). Across these three emotions, performance was better under the control and mask conditions compared with the window condition (all *ps* < 0.001, *ds* ≥ 1.40).

The main effect of orientation was significant for anger and happiness, showing that performance was better in upright than inverted trials. However, performance was similar in upright and inverted trials for surprise. The interactions between orientation and viewing condition did not reach statistical significance for any of these emotions.

## 4. Discussion

By using a gaze-contingent paradigm, the present study explored the effect of foveal and extrafoveal information on emotion recognition. Observers were asked to identify facial emotions in full view, which allows for both foveal and extrafoveal information (i.e., control condition), when only foveal information was available (i.e., window condition), and when only extrafoveal information was available (i.e., mask condition). Overall, the results show that for upright face performance was similar under the control and mask conditions. However, inverted face performance was better under the control condition compared with the mask or window condition and under the mask compared with the window condition. These patterns of results are similar to those found in face identification tasks (Van Belle et al., 2010b). Interestingly, when the type of emotion was included in the analysis, we found that for happy, angry, and surprised faces, the orientation effect was independent from the viewing condition. In other words, for these emotions, performance was higher under the control and mask conditions compared with the window condition, with this effect being equivalent for upright and inverted faces. If it is assumed that the window and mask conditions disrupt holistic and featural processing, respectively (e.g., Van Belle et al., 2010b), this effect reflects that happy, angry, and surprise facial emotions likely rely on a combination of both featural and holistic processing. In fact, previous research has shown that perceiving the mouth is enough to identify happy faces (Calvo et al., 2014).

Although we found that isolating foveal information impaired emotion recognition across different emotions, isolating extrafoveal information had more variable effects, impairing only the recognition of sad faces. Thus, our results suggest that at normal conversation distance and when faces are presented centrally, extrafoveal information is generally sufficient for emotion recognition. Our results are in agreement with previous studies in patients with age-related macular degeneration, a major vision impairment affecting central vision. Although these patients presented substantial problems in detecting whether a face had an expression or not, despite their remarkable problems in central vision, they were still able to identify emotions with a level of performance that was close to that of age-matched controls (Boucart et al., 2008). However, our findings contrast with previous reports that found that all face emotions except happy faces were impaired when presented parafoveally (Calvo et al., 2014). Two different reasons could explain these differences. First, Calvo et al. (2014) analysed accuracy—measured with A’ (Snodgrass & Corwin, 1988)—and RTs separately, and the advantage for parafoveally presented happy faces was only found in accuracy. The problem of this approach is that it does not account for potential speed–accuracy trade-offs. To address this issue, in our study, we used the RCS as the dependent variable to avoid such trade-offs, as it combines reaction times and accuracy. Second, and perhaps more importantly, Calvo et al. (2014) manipulated parafoveal information by presenting faces eccentrically from the centre of the screen. This approach confounds face position and the type of information and prevents observers from processing faces by using face-specific mechanisms (Goren & Wilson, 2006).

Our results are not without limitations. First, although our findings suggest that extrafoveal information suffices for emotion recognition, the individual contributions of parafoveal and peripheral information remain unknown, as our manipulation disrupted both. Based on previous findings (Calvo et al., 2010, 2014), we tentatively suggest that parafoveal information is more important than peripheral information at a normal conversation distance, as more facial features would fall within the former. This issue, however, could be experimentally tested in the future by adapting the gaze-contingent paradigm to individually disrupt either parafoveal or peripheral information. In addition, while facial expressions are universally identified, research has shown important cultural differences in general visual strategies. In fact, it has been suggested that compared with Western individuals, people from Asian backgrounds exhibit a stronger bias toward a more global distribution of visual attention (Ji et al., 2000; McKone et al., 2010), and these differences also extend to the processing of faces (Blais et al., 2008, 2021). Thus, it is possible that the effects of foveal and extrafoveal information differ across cultures. In this study, all participants were of Southeast Asian origin. Given their tendency for more global information processing, it is possible that the mask effect may be reduced compared with Western participants, while the window effect could be amplified. Future research could investigate this by employing a gaze-contingent paradigm to directly compare these cultural groups. Finally, while common in cognitive psychology research, we acknowledge that using static, cropped facial images may limit the ecological validity of our findings.

In conclusion, the results of this study indicate that at a normal conversation distance, isolating extrafoveal information has minimal impact on the recognition of different emotions. However, isolating foveal information significantly impairs identification. These findings suggest that extrafoveal information is generally sufficient for accurate emotion recognition.

## Figures and Tables

**Table 1:** Separate ANOVAs for each emotion.

Emotion	Orientation	Viewing Condition	Orientation × Viewing Condition
Fear	F = 20.28, *p* < 0.001, η^2^_p_ = 0.42	F = 69.47, *p* < 0.001, η^2^_p_ = 0.71	F = 4.44, *p* < 0.05, η^2^_p_ = 0.13
Sadness	F = 29.79, *p* < 0.001, η^2^_p_ = 0.516	F = 141.29, *p* < 0.001, η^2^_p_ = 0.835	F = 10.78, *p* < 0.001, η^2^_p_ = 0.27
Happiness	F = 5.03, *p* < 0.05, η^2^_p_ = 0.15	F = 122.80, *p* < 0.001, η^2^_p_ = 0.81	F = 0.78, *p* = 0.46
Surprise	F = 0.01, *p* = 0.97	F = 85.64, *p* < 0.001, η^2^_p_ = 0.75	F = 2.82, *p* = 0.06
Anger	F = 46.66, *p* < 0.001, η^2^_p_ = 0.62	F = 52.86, *p* < 0.001, η^2^_p_ = 0.65	F = 2.84, *p* = 0.09

## Data Availability

The original data presented in the study are openly available in OSF at 10.17605/OSF.IO/UX63E.
